# Case Report: Breast metastasis from biliary tract cancer: a rare case and literature review

**DOI:** 10.3389/fonc.2026.1705918

**Published:** 2026-02-26

**Authors:** Yujun Tong, Yuzhu Ji, Zhen Zhang, Xiaohong Zhang, Zhuowei Tang

**Affiliations:** 1 Department of Breast Center, Mianyang Central Hospital, School of Medicine, University of Electronic Science and Technology of China, Mianyang, China; 2 Department of Pathology, Mianyang Central Hospital, School of Medicine, University of Electronic Science and Technology of China, Mianyang, China

**Keywords:** biliary tract cancer, breast metastasis, case report, diagnosis, epithelial-mesenchymal transition (EMT), immunohistochemistry, mucinous adenocarcinoma

## Abstract

Breast metastasis from biliary tract cancer (BTC) is an extremely rare clinical occurrence. We report the case of a 59-year-old woman who was incidentally found to have a hepatic mass, leading to a comprehensive workup prompted by progressive chest discomfort. Clinical examination revealed a firm left breast with nipple retraction and a palpable axillary mass. Imaging confirmed a non-mass enhancement lesion in the left breast along with extensive metastases to the liver, lungs, bone, and mediastinal lymph nodes. Core needle biopsies of the breast and abdominal wall lesion showed morphologically similar adenocarcinoma. Immunohistochemistry (IHC) was instrumental in the diagnosis, demonstrating positivity for CK19 and IMP3, focal positivity for CDX2, and a high Ki-67 index (80%), while key breast markers (GATA3, ER, PR, and HER2) were all negative. This immunoprofile strongly supported a biliary origin. The patient was diagnosed with advanced BTC with multiple metastases, including to the breast. She received supportive care and was referred for systemic therapy evaluation. This case highlights that BTC breast metastasis can mimic primary breast carcinoma, underscoring the critical role of pathology and IHC in achieving an accurate diagnosis, which is essential for guiding appropriate, individualized treatment and avoiding unnecessary surgery.

## 1 Introduction

Biliary tract carcinoma (BTC), including cholangiocarcinoma and gallbladder carcinoma, is a highly aggressive malignancy characterized by early local invasion and a strong propensity for distant metastasis. BTC most commonly metastasizes to the liver, followed by regional and distant lymph nodes, peritoneum, lungs, and bone; less frequent sites include the small bowel and other abdominal organs. In contrast, metastatic involvement of the breast from extramammary malignancies is rare, accounting for approximately 0.1% to 5% of all malignant breast tumors, with substantial variability across studies due to differences in diagnostic criteria, patient populations, and study design (1–5). Within this already uncommon category, breast metastasis originating from BTC is exceptionally rare. To date, only a limited number of such cases have been reported worldwide, and precise epidemiological quantification remains unavailable. Given the aggressive biological behavior and heterogeneous metastatic patterns of BTC, rare metastatic sites may occasionally be observed, although their true incidence cannot be reliably estimated (6). Clinically and radiologically, BTC breast metastases may closely mimic primary breast carcinoma, posing significant diagnostic challenges and potentially leading to inappropriate surgical management or delays in initiating appropriate systemic therapy if not accurately recognized. While existing reports have largely focused on clinical presentation and patient outcomes, the biological mechanisms underlying BTC metastasis to the breast remain poorly understood. In this context, we present a rare case of BTC with breast metastasis and review the relevant literature, with particular emphasis on clinicopathological features, diagnostic pitfalls, and potential underlying biological mechanisms.

## 2 Case report

A 59-year-old woman was admitted to the hospital after an incidental hepatic mass was discovered during a routine health examination. She also reported progressive left chest wall pain and an unintentional weight loss of 5 kg over the previous three months. Her medical history was notable for a prior intrahepatic bile duct stone surgery. There was no personal or family history of malignancy.

On physical examination, the left breast was firm with nipple retraction and overlying skin thickening. A hard, immobile, and painless lymph node measuring approximately 3 cm was palpable in the left axilla. Abdominal examination was otherwise unremarkable.

**Figure 1:**
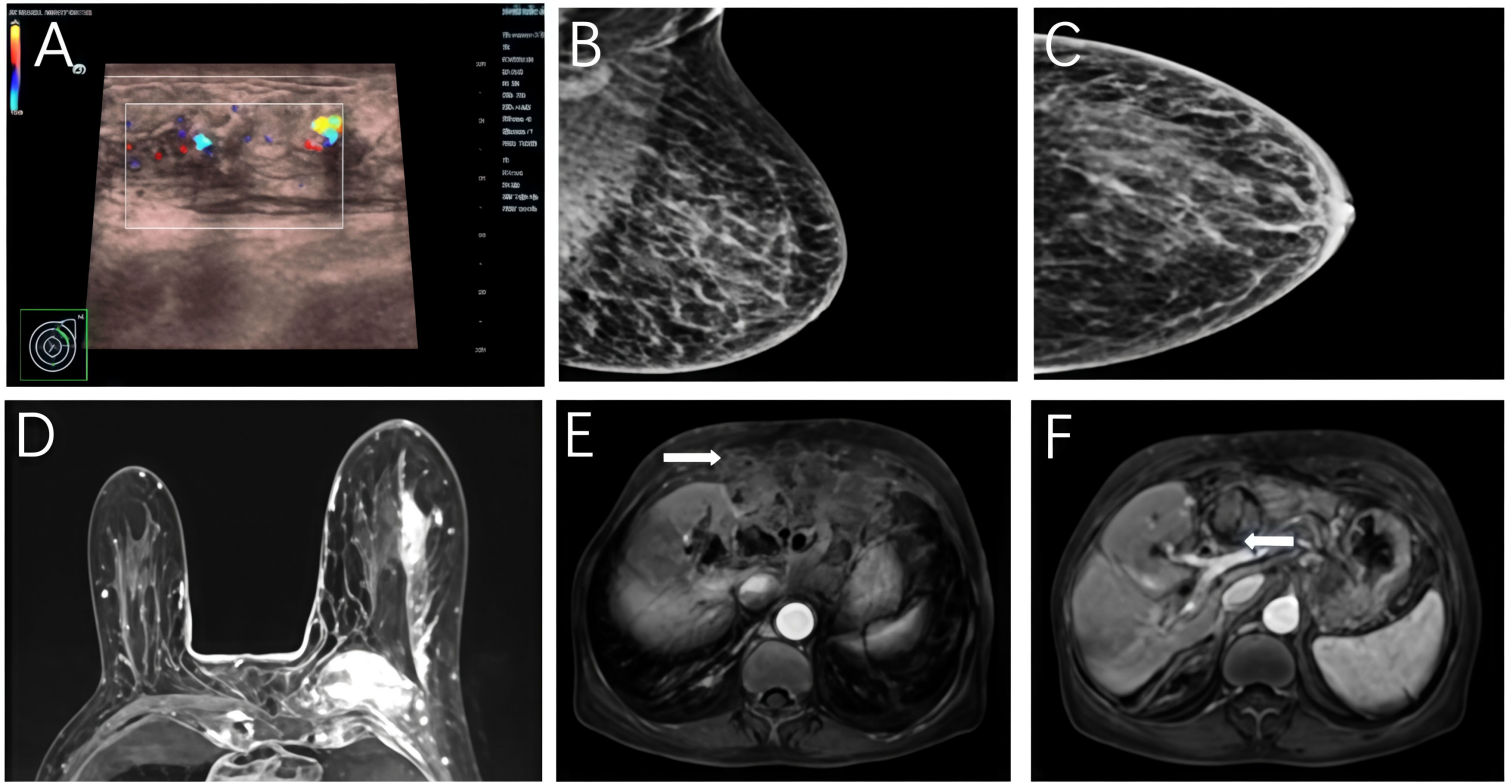
Imaging findings of the patient. **(A)**, Breast ultrasound reveals an irregular hypoechoic lesion with increased internal vascularity. **(B, C)**, Mammography **(B)**, mediolateral oblique view; **(C)**, craniocaudal view) shows diffuse architectural distortion and increased parenchymal density in the left breast with ipsilateral axillary lymph node enlargement. **(D)**, Contrast-enhanced breast MRI demonstrates extensive non-mass enhancement in the upper outer quadrant of the left breast. **(E, F)**, Abdominal contrast-enhanced MRI reveals an irregular hepatic mass with intrahepatic bile duct dilatation **(E)** and abutment of the mass to the chest wall and pericardium, suggesting local invasion **(F)**.

Following physical examination, first-level breast imaging was performed. Breast ultrasound revealed an irregular hypoechoic area in the left breast with increased internal vascularity(**Figure 1A**). Mammography demonstrated architectural distortion and increased parenchymal density of the left breast, accompanied by mild nipple retraction and enlarged left axillary lymph nodes. Given the diffuse nature of the abnormality and the absence of a clearly defined focal lesion requiring immediate biopsy. The mammographic assessment was categorized as BI-RADS 0, indicating the need for further imaging evaluation (**Figures 1B, C**). Given the inconclusive findings on first-level imaging and the presence of extensive clinical and systemic disease, contrast-enhanced breast MRI was subsequently performed for further lesion characterization and staging. Breast MRI demonstrated extensive non-mass enhancement in the upper outer quadrant of the left breast, measuring 7.6 × 1.7 × 4.7 cm, without a discrete mass. These findings were accompanied by ipsilateral axillary and mediastinal lymphadenopathy, and the examination was classified as BI-RADS 5 (**Figure 1D**).

Abdominal contrast-enhanced MRI showed an irregular hepatic mass (8.4 × 6.2 cm) with intrahepatic bile duct dilation, which was closely abutting the chest wall and pericardium, suggesting local invasion (**Figures 1E, F**). Subsequent chest computed tomography (CT) confirmed left breast thickening, axillary lymphadenopathy, and multiple pulmonary lesions. Bone scintigraphy showed abnormal uptake in the sternum and ribs, consistent with osseous metastases.

**Figure 2:**
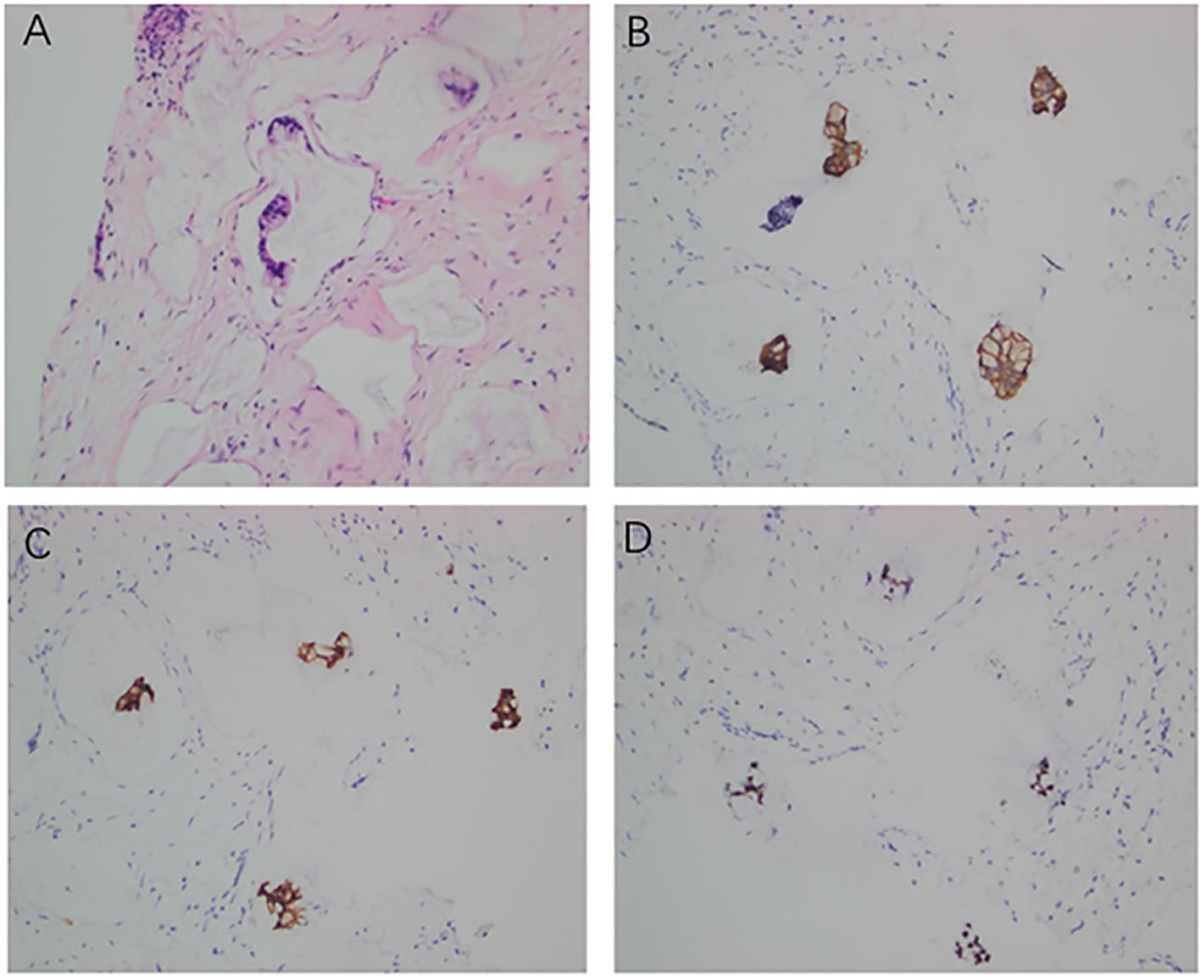
Pathological and immunohistochemical features of the hepatic lesion. **(A)** Adenocarcinoma with abundant mucinous component (H&E, ×200). **(B)** CK19 diffusely positive (IHC, ×200). **(C)** IMP3 positive (IHC, ×200). **(D)** CDX2 focal positive (IHC, ×200).

**Figure 3:**
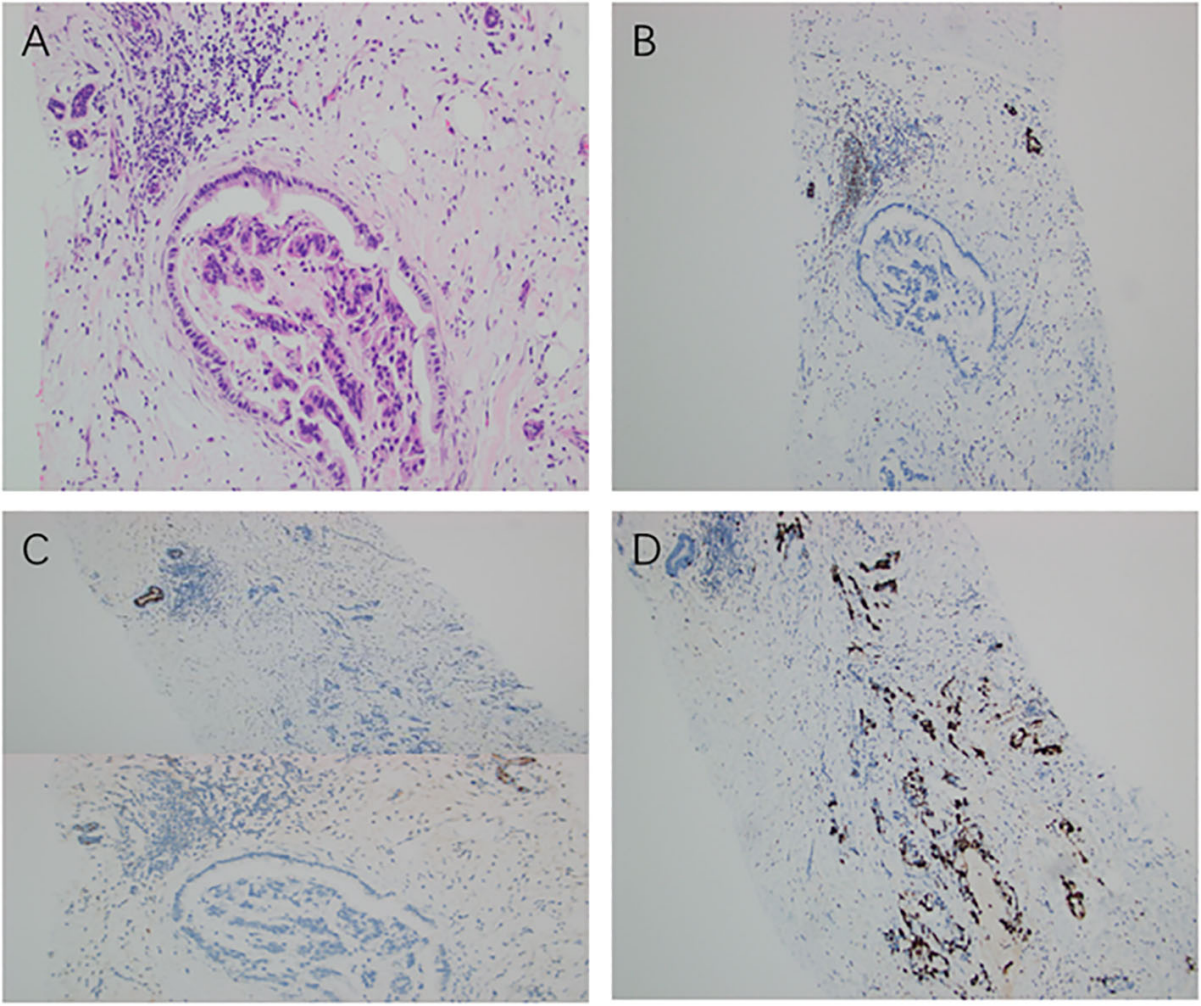
Pathological and immunohistochemical features of the breast lesion. **(A)** Infiltrating adenocarcinoma morphologically similar to the hepatic lesion (H&E, ×200). **(B)** GATA3 negative (IHC, ×100). **(C)** ER/PR negative (IHC, ×200). **(D)** Ki-67 about 80% (IHC, ×200).

Histopathological examination of core biopsies from the abdominal wall, breast mass, and axillary lymph node all showed infiltrating adenocarcinoma with similar mucinous morphology. Immunohistochemical (IHC) analysis was critical for determining the primary origin. The tumor cells demonstrated diffuse positivity for CK19 and IMP3, focal positivity for CDX2, and a high proliferative index (Ki-67 of 80%). Importantly, breast-specific markers, including GATA3, ER, PR, and HER2, were all negative. Furthermore, the tumor cells showed diffuse positivity for E-Cadherin and negativity for P63, CK5/6, CK7, and CK20 (**Figure 2** and **3**). This immunoprofile strongly supported a biliary tract origin rather than a primary breast carcinoma.

Based on the integration of comprehensive clinical, radiological, and histopathological findings, the patient was diagnosed with advanced biliary tract carcinoma (cT4N3M1) with widespread metastases, including to the breast. She received supportive care and was referred to a higher-level hospital for a multidisciplinary evaluation and the initiation of systemic therapy.

## 3 Discussion

Secondary malignancies of the breast are uncommon, and metastasis from BTC is a particularly rare event, with fewer than 15 cases reported in the literature (2, 7–12). As highlighted by our case, these metastases often present as breast lesions or non-mass enhancements, which can easily be mistaken for primary breast carcinoma.

Difficulty in distinguishing breast metastases from primary breast carcinoma remains a clinically relevant challenge and may contribute to inappropriate surgical intervention or delayed initiation of systemic therapy. Potential sources of diagnostic uncertainty include reliance on imaging findings alone, limited immunohistochemical panels, and insufficient consideration of the patient’s overall systemic disease burden. From a practical perspective, accurate differentiation between metastatic lesions and primary breast carcinoma relies on awareness of atypical imaging presentations—such as extensive non-mass enhancement without associated ductal carcinoma in situ—together with comprehensive immunohistochemical profiling and careful clinicopathological correlation. Early multidisciplinary collaboration among radiologists, pathologists, and oncologists further facilitates accurate diagnosis and appropriate treatment selection. With regard to the existing literature, although well-documented cases of biliary tract carcinoma metastasizing to the breast remain exceedingly rare, breast metastases from extramammary malignancies have been reported to pose significant diagnostic challenges and may closely mimic primary breast carcinoma, underscoring the broader clinical relevance of accurate differentiation (9).

Accurate diagnosis hinges on a comprehensive clinicopathological evaluation. Histology and immunohistochemistry are the cornerstones of this process. The immunoprofile in our patient—positive for CK19 and IMP3 and negative for GATA3, ER, PR, and HER2—is highly consistent with the reported characteristics of BTC breast metastases. This unique immunophenotype provides the definitive evidence required to distinguish it from a primary breast cancer. While GATA3 is a sensitive marker for breast carcinoma, its expression is not entirely specific, as weak positivity has been noted in other tumor types (13). In ambiguous cases, molecular profiling with next-generation sequencing (NGS) can confirm the clonal origin of the tumor and identify actionable mutations (e.g., FGFR2 fusions or IDH1 mutations) to guide targeted therapy (14, 15).

The biological mechanisms underlying metastasis of BTC to rare sites such as the breast remain largely speculative. Epithelial–mesenchymal transition (EMT) and microRNA dysregulation have been implicated in BTC dissemination and may contribute to tumor cell plasticity during metastatic spread (16–18). In the present case, preserved E-cadherin expression suggests that the tumor retained epithelial characteristics, supporting the concept of partial or reversible EMT during metastasis. In addition, the “seed and soil” hypothesis provides a conceptual framework for understanding organ-specific colonization. The mucinous morphology observed in this case may facilitate tumor cell survival and implantation at distant sites through mucin-associated protective and adhesive properties (19). These mechanisms are discussed as potential biological context rather than definitive explanations for this rare metastatic presentation.

Given the advanced stage of BTC breast metastasis, systemic therapy remains the cornerstone of management. The standard first-line regimen of gemcitabine plus cisplatin (GEM-CIS) has been shown to improve median overall survival to approximately 11.7 months compared to gemcitabine alone (20). However, the efficacy of this regimen is limited by its associated toxicities and the inevitable development of resistance. Targeted therapies are also emerging for patients with specific molecular alterations. In this context, management of the breast lesion is primarily for diagnostic or palliative purposes, as radical surgery does not improve overall prognosis (7, 11). Multidisciplinary collaboration is therefore essential for accurate diagnosis and the development of a tailored, systemic treatment plan.

## 4 Conclusion

Breast metastasis from biliary tract carcinoma is an exceptionally rare but clinically important entity that can closely mimic primary breast carcinoma and pose challenges in differential diagnosis. This case highlights the necessity of a comprehensive diagnostic approach that integrates clinical, radiological, and histopathological findings, particularly with the use of an extensive immunohistochemical panel. Understanding the potential underlying mechanisms, such as EMT plasticity, miRNA regulation, and mucin-related interactions, may provide insights into the pathogenesis of this unusual presentation. Ultimately, the diagnosis of metastatic BTC to the breast should prompt the immediate initiation of systemic therapy, with multidisciplinary collaboration being vital for optimal patient care.

## Data Availability

The original contributions presented in the study are included in the article/supplementary material. Further inquiries can be directed to the corresponding authors.
